# Occupational exposure and health surveys at metal additive manufacturing facilities

**DOI:** 10.3389/fpubh.2023.1292420

**Published:** 2023-11-20

**Authors:** Maria Assenhöj, Ann-Charlotte Almstrand, Spela Kokelj, Stefan A. Ljunggren, Anna-Carin Olin, Helen Karlsson

**Affiliations:** ^1^Occupational and Environmental Medicine Center in Linköping, Department of Health, Medicine and Caring Sciences, Linköping University, Linköping, Sweden; ^2^Occupational and Environmental Medicine, Sahlgrenska University Hospital, Region Västra Götaland, Gothenburg, Sweden; ^3^Occupational and Environmental Medicine, School of Public Health and Community Medicine, Institute of Medicine, University of Gothenburg, Gothenburg, Sweden

**Keywords:** 3D-printing, additive manufacturing, powder bed fusion, binder jetting, metals, occupational exposure, particle exposure, occupational health

## Abstract

**Introduction:**

Additive manufacturing is a novel state-of-the art technology with significant economic and practical advantages, including the ability to produce complex structures on demand while reducing the need of stocking materials and products. Additive manufacturing is a technology that is here to stay; however, new technologies bring new challenges, not only technical but also from an occupational health and safety perspective. Herein, leading Swedish companies using metal additive manufacturing were studied with the aim of investigating occupational exposure and the utility of chosen exposure- and clinical markers as predictors of potential exposure-related health risks.

**Methods:**

Exposure levels were investigated by analysis of airborne dust and metals, alongside particle counting instruments measuring airborne particles in the range of 10 nm−10 μm to identify dusty work tasks. Health examinations were performed on a total of 48 additive manufacturing workers and 39 controls. All participants completed a questionnaire, underwent spirometry, and blood and urine sampling. A subset underwent further lung function tests.

**Results:**

Exposure to inhalable dust and metals were low, but particle counting instruments identified specific work tasks with high particle emissions. Examined health parameters were well within reference values on a group level. However, statistical analysis implied an impact on workers kidney function and possible airway inflammation.

**Conclusion:**

The methodology was successful for investigating exposure-related health risks in additive manufacturing. However, most participants have been working <5 years. Therefore, long-term studies are needed before we can conclusively accept or reject the observed effects on health.

## 1 Introduction

Additive manufacturing (AM) is the process of building parts from computer-aided designs by joining materials, often layer by layer (1). Metal AM is a novel state-of-the art technology with great potential. It allows for the production of complex structures that are otherwise not possible with traditional subtractive manufacturing processes and offers the advantage of on-demand production, reducing the need for large product inventories (2). Metal AM may be achieved by different techniques that join metal together. These include binder jetting (BJT), which utilizes a liquid wax bonding agent to hold the metal particles together until sintering the component; as well as powder bed fusion (PBF), where a powder is added in sheets and then joined in specific places by a laser, electron beam, or electric arc (1). Furthermore, there is an increasing number of powders or feedstocks available (3). Despite the promises of metal AM techniques, they often utilize potentially harmful metals, including nickel, cobalt, and chromium, which are known to be detrimental to human health (4). Moreover, emission of nano- and submicron particles during AM processes (5, 6) may also have hazardous effect on human health (7). Therefore, there is a need to better understand different occupational settings and their potential to induce harm to the exposed individuals.

There remains a limited amount of literature on exposure and possible health effects in metal AM. A review of exposure assessment and health hazards of particulate matter in metal additive manufacturing has been conducted (8), and recent discussions have focused on implications for risk assessment and management in occupational settings (9). In occupational settings, the respiratory system is the most important exposure target and should be considered first when evaluating potential harm to workers. Lung function is traditionally evaluated by spirometry, and a long-term study using spirometry found a decline in Forced expired volume in the first second (FEV1) and the FEV1/Forced expiratory vital capacity (FVC) ratio due to occupational exposure to metals (10). Spirometry can be complemented with additional lung function tests to provide a deeper insight into respiratory health. For example, fractional exhaled nitric oxide (FeNO) can be used as a biomarker of respiratory tract inflammation, not only in asthma but also in various other respiratory diseases often linked to work-related factors. FeNO provides a valuable tool to monitor the effect of occupational exposures on respiratory health, moreover, it is more sensitive than spirometry (11). Additionally, impulse oscillometry (IOS) provides important information regarding the small airways, which are often involved early in the course of the diseases. IOS can detect increased airway resistance before the onset of symptoms and abnormal spirometry (12). Moreover, IOS is more sensitive than spirometry for detecting small airway disease in asthma and post-environmental exposure (13, 14). Breath analysis can also be used to retrieve biological samples from the small airways, by sampling of exhaled endogenous particles that are formed from the respiratory tract lining fluid. Analysis of particles in exhaled air can help detect early changes directly or indirectly related to ambient air and occupational exposure, as well as airway diseases (15–17). Changes in the lung surfactant composition, including phosphatidylcholine species and surfactant protein A, have been observed in exhaled particles from smokers and subjects with asthma (18, 19).

Individual exposure to metals can be assessed through the analysis of metals in urine, which can be a valuable tool when investigating possible adverse health effects (20–23). However, measurements of urine metals should be combined with clinical effect markers of target organs before drawing conclusions regarding the effects of metal AM. Metal exposure can effect various organs, such as kidney (24), liver (25, 26), and the cardiovascular system (27, 28). Functional status of these organs can be assessed using common clinical markers including liver markers aspartate aminotransferase (ASAT), alanine aminotransferase (ALAT), and alkaline phosphatase (ALP) in blood, as well as the urine marker of alpha-1-microglobulin (α-1-M) that could identify renal tubular damage (29). In routine healthcare, and studies concerning risk factors of cardiovascular health, apolipoprotein A-I (ApoA-I) and apolipoprotein B (ApoB) are commonly evaluated (30–32). ApoA-I, present on high-density lipoprotein, protects against cardiovascular disease due to the reverse cholesterol transport. Moreover, ApoA-I has important functions in the immune system (33, 34). Other biomarkers associated with cardiovascular disease risk include the acute phase protein serum amyloid A1 (SAA1) and the antioxidant protein serum paraoxonase/arylesterase 1 (PON1), both situated on high-density lipoprotein as well (35).

We have earlier shown that virgin metal AM powder, when used in powder bed fusion laser beam (PBF-LB) machinery, changes to include a large bulk of smaller particles that may constitute different risks to the AM machine user (AMMU) (4). Furthermore, we have thoroughly investigated AMMU at a specific site with serial production of AM components, examining their exposure, biological markers, and effects in the nasal mucosa (5, 32, 36). However, these earlier studies were limited to a single AM technique, two different AM powders, and a smaller number of AMMUs. Therefore, in the current study our aim was to further explore our previous investigation methodology and findings in a larger cohort of AMMU and at different AM facilities using various AM techniques and feedstock materials.

Herein, we report emission data from both BJT and PBF that use a range of different metal powder as feedstock. We have furthermore investigated the AMMU's lung functions, urinary metals, and the aforementioned effect markers to explore potential health effects that may arise due to metal AM.

## 2 Materials and methods

### 2.1 The AM process chain

**Figure 1:**
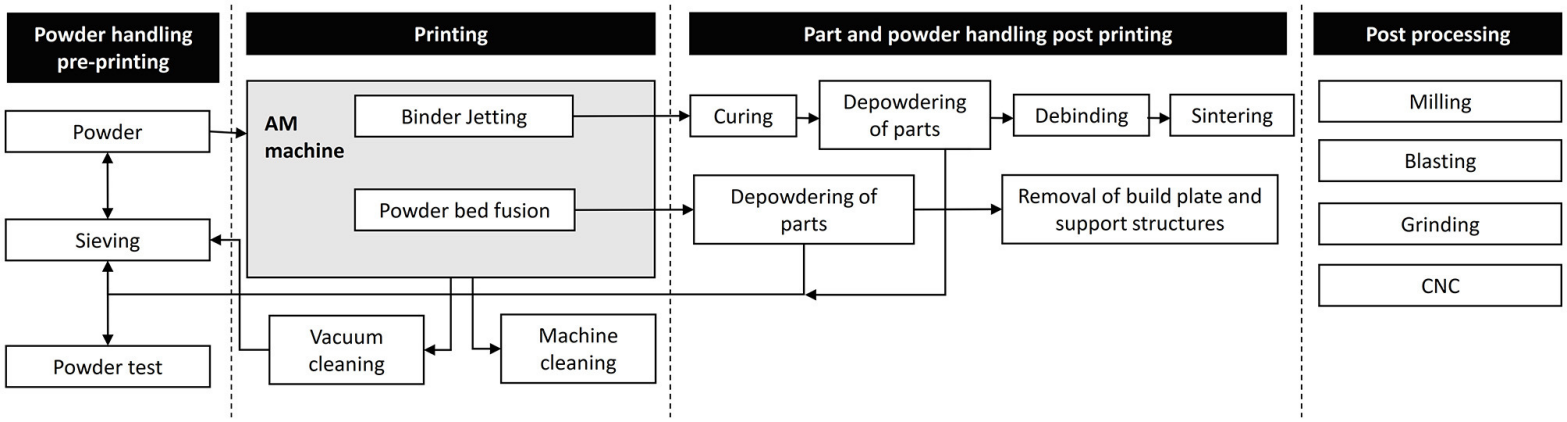
Additive manufacturing (AM) process chain of binder jetting and powder bed fusion.

The AM process chain from powder to the finished part includes several steps. Figure 1 gives an overview of the process chain of the techniques presented in this paper, BJT and PBF. The printing process itself takes place in the AM machine, where the part is built layer by layer. BJT joins materials by selectively adding a liquid bonding agent to a metal powder bed to form the part. Similarly, PBF joins materials by selectively heating the metal powder using either a laser beam (PBF-LB) or an electron beam (PBF-EB). Both BJT and PBF result in a box of powder (job-box) with the printed part embedded in non-melted/liquid bonded powder. When the term printing is used herein, it includes the manual tasks performed by the AMMU, such as preparation of the AM machine, powder refill, taking out the job-box, and machine cleaning. After printing, the so-called green body from BTJ is hardened in an oven at roughly 200°C for several hours so they can be removed safely from the printing bed. This process is called curing. Then, the unbound powder is removed, and printed parts are subjected to de-binding, which partially removes the binding agent. Finally, the parts are sintered in a furnace at roughly 90% of the melting temperature of the alloy for 2–6 h, which consolidates the metal and burns away the remaining binding agent. PBF printed parts can be directly depowdered but usually requires subsequent removal from the build plate and support structures by sawing or other processing. There are several approaches to the process of depowdering varying from manually handling in a fume hood or depowdering stations to more automated systems. The latter sometimes require that an open job-box is moved between printer and the depowdering unit by the AMMU, whereas some can have an enclosed job-box which can be moved by forklift to the depowdering unit. Unused powder from the powder bed and excess powder from the printer is recirculated but it needs to be processed by sieving to ensure the desired size distribution. Emptying containers, such as vacuum cleaners and overflow chambers, during recirculation and sieving of powder is included in the term sieving in our measurement data. Powder tests are routinely performed to evaluate virgin powder, used powder, and powder blends. These tests use small volumes of powder. Post-processing is performed on the parts after the finished AM build cycle to get the final product. Post-processing includes techniques such as milling and grinding, either by handheld tools or computer numerical control (CNC) machines, and blasting.

Since all handling of metal powders entails a risk of exposure, there are possible high-exposure situations associated with powder manufacturing. Here, single powder processing steps such as powder testing and sieving can be a full-time job for one operator, thus leading to continuous chance of exposure to powders. Furthermore, metal feedstock is melted using high temperature ovens and operators could thus be highly exposed to metal fumes.

### 2.2 Exposure measurements

#### 2.2.1 Sampling locations

Exposure measurements of metals were performed at eight different companies/facilities. Investigated printing techniques included PBF-LB, PBF-EB, and BJT. The techniques utilize various metal powders including iron-, nickel-, cobalt-, or titanium-based powders (Table 1).

**Table 1:** Measurements in this study were performed on these printing techniques and powders.

**Technique**	**Printer manufacturer**	**Printer type**	**Iron-based powder**	**Nickel-based powder**	**Titanium-based powder**	**Cobalt-based powder**	**Other**
LB-PBF	EOS	M290, M280, M400-4	√	√	√	–	–
LB-PBF	SLM solutions	SLM125HL, SLM280	√	√	–	–	–
LB-PBF	Xact Metals	XM200C	√	–	–	–	–
EB-PBF	Arcam EBM	Q10Plus, SpectraH	–	√	√	√	–
BJT	Digital metal	DM P2500	√	√	√	–	–
BJT	ExOne	X1 25Pro, M-Flex	–	–	–	–	√

BJT, binder jetting; PBF-LB, powder bed fusion-laser beam; PBF-EB, powder bed fusion-electron beam.

#### 2.2.2 Gravimetric and metal analyses

Sampling of total and inhalable dust was performed using an open-faced cassette and an IOM-sampler (SKC Ltd, Dorset, UK), respectively, in accordance with Swedish standards (37, 38). Both samplers were used with a pre-weighed 25 mm membrane filters with a pore size of 0.8 μm, and operated with an airflow of 2 L/min. Airflow rate of all samplers were measured using calibrated flow meters before and after sampling. Personal sampling was performed by placing samplers in the breathing zone, while stationary samplers were placed at locations of interest for emission or operator exposure. Sampling of total and inhalable dust on filters were analyzed gravimetrically for particulate mass. Subsequently, metal analyses were performed, similar to a previously described method (39), on all total dust filters and on inhalable dust filters with a dust concentration above 0.3 mg/m^3^ by inductively coupled plasma mass spectrometry (iCAP™-Q; Thermo Fisher Scientific, Waltham, MA, USA) at the Occupational and Environmental Medicine Analytical Laboratory at Linköping University Hospital. A report limit of 0.01 μg per sample was used.

Inhalable dust containing titanium-based alloys was analyzed by ICP-MS (iCAP™-Q; Thermo Fisher Scientific) at the Occupational and Environmental Medicine Analytical Laboratory at Örebro University Hospital. The titanium method is based on SS-ISO 15202-2 and 15202-3 standards (40, 41) with addition of hydrofluoric acid during sample preparation. The method had a limit of quantification of 0.5 μg/sample.

#### 2.2.3 Particle measurements

Particle measurements were performed using two types of handheld particle counting instruments: Lighthouse 3016 IAQ (Lighthouse Worldwide Solutions, CA, USA) and Philips Aerasense Nanotracer (Phillips, Best, the Netherlands). The Lighthouse 3016 IAQ is an optical particle counting instrument that simultaneously measures six different particle sizes (0.3, 0.5, 1, 2.5, 5, and 10 μm). The instrument provides the number of particles per m^3^ and an approximate mass using the presumption that all particles are spherical and a set density of 5 g/ml. The Philips Aerasense Nanotracer measures ultrafine particles between 10 and 300 nm. The measuring technique is based on diffusion charge, wherein particles entering the machine are electrically charged and then the total charge is measured. The Nanotracer provides information about particle concentration (particles/cm3) and average particle size (nm).

### 2.3 Health examinations

Health examinations consisted of a questionnaire, blood and urine sampling, as well as performing spirometry. A subset of the participants underwent further lung function tests.

#### 2.3.1 Study participants

Voluntary study participants working in the AM environments were recruited from the available workforce at the participating companies. Controls were unexposed office personnel recruited from the same companies.

#### 2.3.2 Questionnaire

Participants answered a questionnaire with nine questions concerning symptoms known to be related to indoor air problems adapted from the MM040NA questionnaire (42). The questionnaire included questions regarding asthma, allergies, and smoking habits. A translated copy of the questionnaire instrument (used original in Swedish) is available in Supplementary Experimental Procedures.

#### 2.3.3 Urine samples

Acid-cleaned sampling tubes were used for collection of urine samples at the start and at the end of the workweek following a clinical protocol used to reduce contamination risk. Samples were kept refrigerated until arrival at the laboratory where they were frozen (−20°C) until analysis. All samples had measurements for specific gravity, creatinine, and the clinical marker for renal function α-1-M. Creatinine and α-1-M were analyzed at Clinical Chemistry Laboratory, Linköping University Hospital, Sweden. The chosen metal analyses in urine samples from AMMU and controls depended on the powders used at the workshops. U-metal was assessed by ICP-MS (iCAP™ Q; Thermo Fisher Scientific) at the Occupational and Environmental Medicine Laboratory, Linkoping University Hospital, Sweden. Analysis of U-titanium was performed by ICP-SFMS at ALS Scandinavia, Umeå, Sweden. Report limit of the analysis was 1 μg/L (20 nmol/L). If both specific gravity and creatinine levels were below the recommended lower limit (0.010 kg/L and 0.3 g/L, respectively) in a sample, both Monday and Friday urine samples from the participant were excluded (*n* = 7).

#### 2.3.4 Blood samples

At the end of the workweek, blood was collected into lithium heparin tubes, centrifuged at 1200 G for 12 min, and the plasma transferred to new tubes. All samples were directly frozen in −20°C until analysis. Three clinical markers for hepatic function were assessed: ASAT, ALAT, and ALP, along with two clinical markers for cardiovascular status: ApoA-I and ApoB. All clinical analyses were performed at the Clinical Chemistry Laboratory, Linköping University Hospital, Sweden. Analyses for SAA1 and PON1 were performed by ELISA and an enzyme activity assay previously described in Ljunggren et al. (32).

#### 2.3.5 Lung/airway tests

Spirometry was performed using a handheld PC-based spirometer (SpiroUSB, Becton, Dickinson and Company, US), according to American Thoracic Society (ATS) and European Respiratory Society (ERS) guidelines (43). Calibration was performed daily, and measurements were performed in triplicate. Both FEV1 and FVC were evaluated. The percentage of predicted FEV1 and FVC as well as the FEV1/FVC ratio were compared against sex-specific Swedish reference materials (44, 45).

IOS was performed using a Jaeger Masterscreen system (CareFusion, Hochberg, Germany), according to ATS/ERS recommendations (46). Mean values of resistance at 5 Hz (R5 Hz) and 20 Hz (R20 Hz), frequency dependence of resistance (R5–20 Hz) and the reactance area (AX) were calculated and expressed as both percent predicted and *z*-scores, according to Kjellberg et al. (47). These parameters are known to increase in patients with airway diseases (13, 48). The critical value for a significance level of 0.05 is a *z*-score < -1.64 or >=1.64.

FeNO was measured with a chemiluminescent analyzer (NIOX VERO^®^ instrument AER-12-1850, Aerocrine AB, Stockholm, Sweden) at an expiratory flow of 50 ml/s. The measurements were in accordance with ATS/ERS recommendations (49). FeNO levels of < 25 of parts per billion (ppb) is considered normal, 25–50 ppb as intermediate, and >50 ppb high (50).

Collection and analysis of particles, surfactant protein A (SP-A), albumin, and phosphatidylcholine (PC) lipids in exhaled breath (PEx samples) were performed by the same method as in Almstrand et al. (17).

#### 2.3.6 Statistical analyses

Statistical analyses were performed in IBM^®^ SPSS^®^ Statistics V27, New York, USA. Chi-square and Fishers exact tests were used to investigate differences in group distribution of sex, age, body mass index, smoking status, and questionnaire answers. Odds ratio for questionnaire answers was determined using univariate logistic regression. Shapiro-Wilk test for normality showed that continuous variables were not normally distributed. Comparison between controls and exposed were analyzed using Mann–Whitney *U*-test and Wilcoxon signed-rank test was used to check for differences between Monday and Friday samples. For α-1-M, values below the limit of detection (LOD, 6.35 mg/L) were imputed with LOD√2 (4.49 mg/L), a similar approach has previously been described for α-1-M (51).

## 3 Results

### 3.1 Exposure measurements

#### 3.1.1 Gravimetric and metal analyses

**Figure 2:**
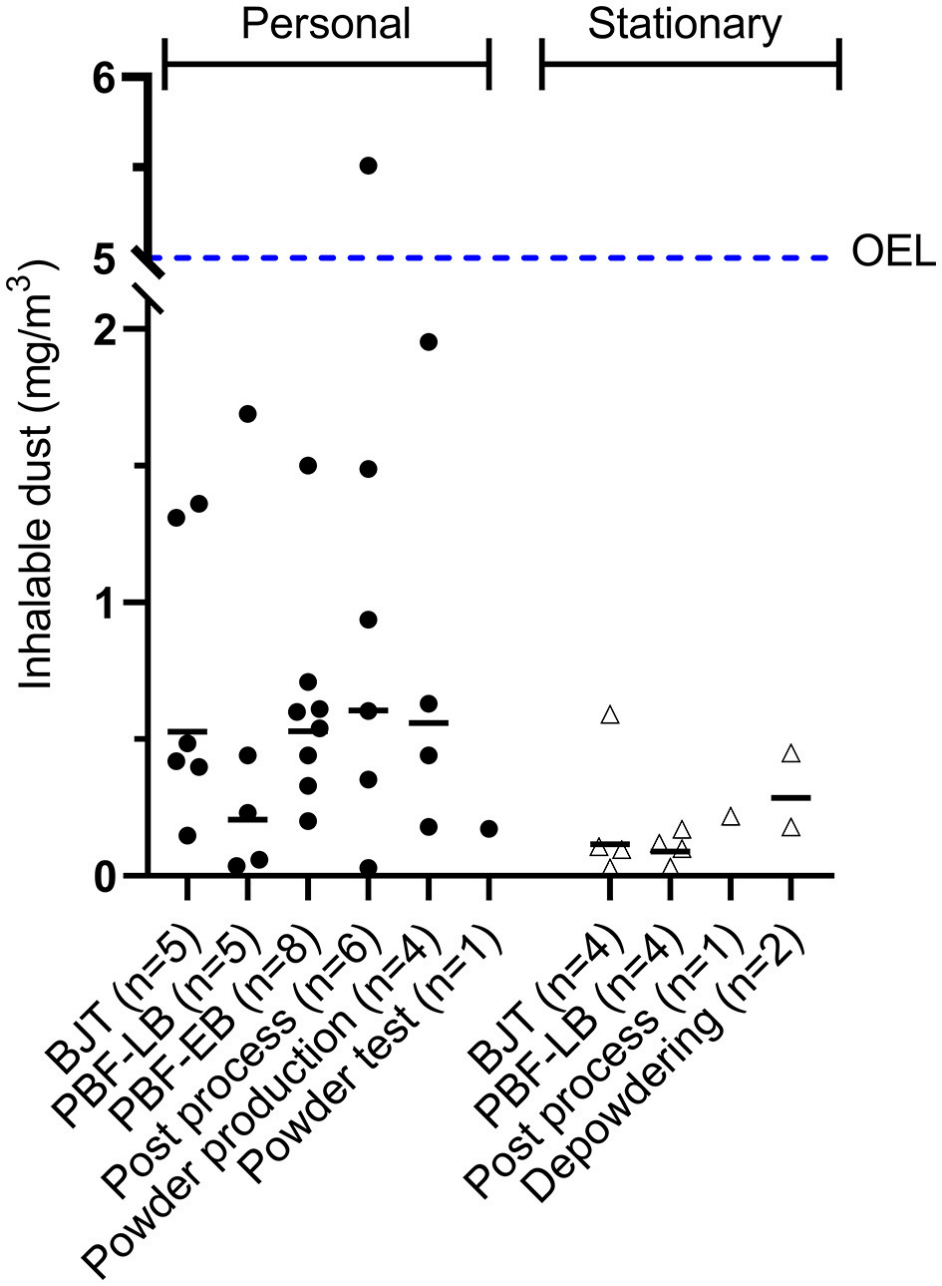
Inhalable dust in air from personal and stationary sampling. The dotted line is the Swedish occupational exposure limit (OEL) for inhalable dust (5 mg/m^3^). BJT, binder jetting; PBF-LB, powder bed fusion-laser beam; PBF-EB, powder bed fusion-electron beam.

Sampling of total and inhalable dust were performed for comparison with current Swedish time-weighted average occupational exposure limits (OELs) for dust and metals. Both total and inhalable dust fractions were collected simultaneously because most metals had their OEL in the total dust fraction (in Sweden). However, for inorganic dust, cobalt, and manganese the OELs were in the inhalable dust fraction. The levels of inorganic dust varied between AM processes and companies, with significantly higher levels of dust in personal samples as compared to stationary samples (*p* < 0.01), Figure 2.

Depending on the materials used at the facilities, either titanium or multiple metal analyses were performed. Multiple metal analyses were performed for 26 and 19 total and inhalable dust samples, respectively, Table 2. Most of the chromium, cobalt, manganese, molybdenum, and nickel levels were below 1%−6% of their respective OEL. However, greater cobalt levels were observed in a BJT printer using an alloy with high cobalt concentrations. Herein, personal and stationary sampling resulted in levels of 11%−35% and 10%−25%, respectively, of cobalt OEL in inhalable dust fraction (0.02 mg/m^3^).

**Table 2:** Air concentrations of metals.

	**AM process**	***n***	**Chromium μg/m^3^**	**Cobalt μg/m^3^**	**Iron μg/m^3^**	**Manganese μg/m^3^**	**Molybdenum μg/m^3^**	**Nickel μg/m^3^**
**Inhalable dust fraction**								
**Swedish OEL/ACGIH**^®^ **threshold limit values**			**–/–**	**20/20**	**–/–**	**200/100**	**–/10,000**	**–/–**
Personal sampling	BJT	5	0.71 (0.44–2.07)	0.17 (< 0.01–7.06)	0.90 (0.25–2.93)	0.04 (0.01–0.12)	0.05 (0.03–0.14)	0.29 (0.04–14.3)
	PBF-LB	2	1.45 (0.72–2.94)	0.09 (0.02–0.36)	2.93 (1.94–4.41)	0.09 (0.06–0.11)	0.19 (0.02–1.53)	2.12 (0.49–9.17)
	Post process	5	0.98 (0.05–6.75)	0.09 (0.01–0.99)	3.10 (0.13–140)	0.05 (< 0.01–1.47)	0.11 (0.01–0.88)	1.24 (0.15–5.23)
	Powder production	3	3.96 (2.64–6.79)	0.22 (0.10–1.18)	16.8 (9.24–24.1)	0.72 (0.20–7.65)	0.55 (0.14–2.15)	6.60 (3.90–14.0)
Stationary sampling	BJT	2	0.39 (0.29–0.51)	0.94 (0.17–5.18)	0.91 (0.36–2.31)	0.03 (0.02–0.04)	0.02 (0.02–0.02)	0.04 (0.03–0.06)
	PBF-LB	2	0.04 (0.01–0.19)	< 0.01 (< 0.01–0.04)	0.47 (0.34–0.64)	0.01 (< 0.01–0.01)	< 0.01 (< 0.01–0.01)	0.01 (< 0.01–0.16)
**Total dust fraction**								
**Swedish OEL/ACGIH**^®^ **threshold limit values**			**500/500**	**-/-**	**–/–**	**–/–**	**10,000/–**	**500/1,500** ^*^
Personal sampling	BJT	4	0.25 (0.09–1.29)	0.03 (< 0.01–1.39)	0.51 (0.17–1.44)	0.02 (0.01–0.07)	0.01 (< 0.01–0.09)	0.35 (0.05–8.77)
	PBF-LB	6	0.18 (0.08–0.50)	0.02 (< 0.01–1.62)	0.60 (0.05–13.4)	0.03 (0.01–0.08)	0.01 (< 0.01–0.82)	0.23 (0.02–5.06)
	Post process	6	0.63 (0.23–7.42)	0.04 (< 0.01–0.72)	2.18 (0.07–123)	0.04 (< 0.01–1.08)	0.10 (0.04–0.95)	0.65 (0.09–4.42)
	Powder production	4	1.47 (0.96–2.69)	0.06 (0.02–0.48)	3.42 (2.05–5.41)	0.72 (0.07–15.6)	0.10 (0.05–0.42)	1.50 (0.92–3.75)
	Powder test	1	0.29	0.01	0.88	3.67	0.06	0.54
Stationary sampling	BJT	2	0.04 (0.01–0.12)	< 0.01 (< 0.01–0.03)	0.02 (< 0.01–0.25)	0.01 (0.01–0.01)	< 0.01 (< 0.01– < 0.01)	0.06 (0.02–0.21)
	PBF-LB	2	0.13 (0.13–0.13)	< 0.01 (< 0.01– < 0.01)	< 0.01 (< 0.01– < 0.01)	0.01 (0.01–0.01)	< 0.01 (< 0.01– < 0.01)	0.01 (0.01–0.01)
	Post process	1	0.01	< 0.01	0.01	< 0.01	< 0.01	0.03

Inhalable and total dust fractions were collected using personal or stationary sampling using inhalable dust samplers (IOM) for inhalable fraction or open-faced cassettes for total dust fraction followed by multiple metal analysis by ICP-MS.

Values are geometric mean (min-max). The bold values are indicate the limit/reference values stated in the left-most column of the tables.

OEL, occupational exposure limit; ACGIH, American Conference of Governmental Industrial Hygienists; BJT, binder jetting; PBF-LB, powder bed fusion-laser beam.

Metal analyses were performed on all total dust filters and on inhalable dust filters with a dust concentration above 0.3 mg/m^3^.

^*^Insoluble nickel, 200 **μg**/m^3^ if soluble.

Inhalable dust and subsequent titanium analysis were analyzed at a PBF-EB facility through eight personal samplings and two stationary samplings, Table 3. Inhalable dust levels in the personal samplings were ~12% of the OEL for the inhalable dust fraction (5 mg/m^3^). Titanium levels were not determined in the stationary samples because of low dust levels (< 0.1 mg/m^3^). There are no OELs for titanium in Sweden.

**Table 3:** Inhalable dust and titanium from personal sampling (*n* = 8).

**Inhalable dust (mg/m^3^)**	**Titanium (μg/m^3^)**	**Titanium (% of inhalable dust)**
0.53 (0.20–1.53)	37.0 (5.78–143)	6.97 (1.30–16.0)

Values are geometric mean (min–max).

#### 3.1.2 Particle measurements

**Figure 3:**
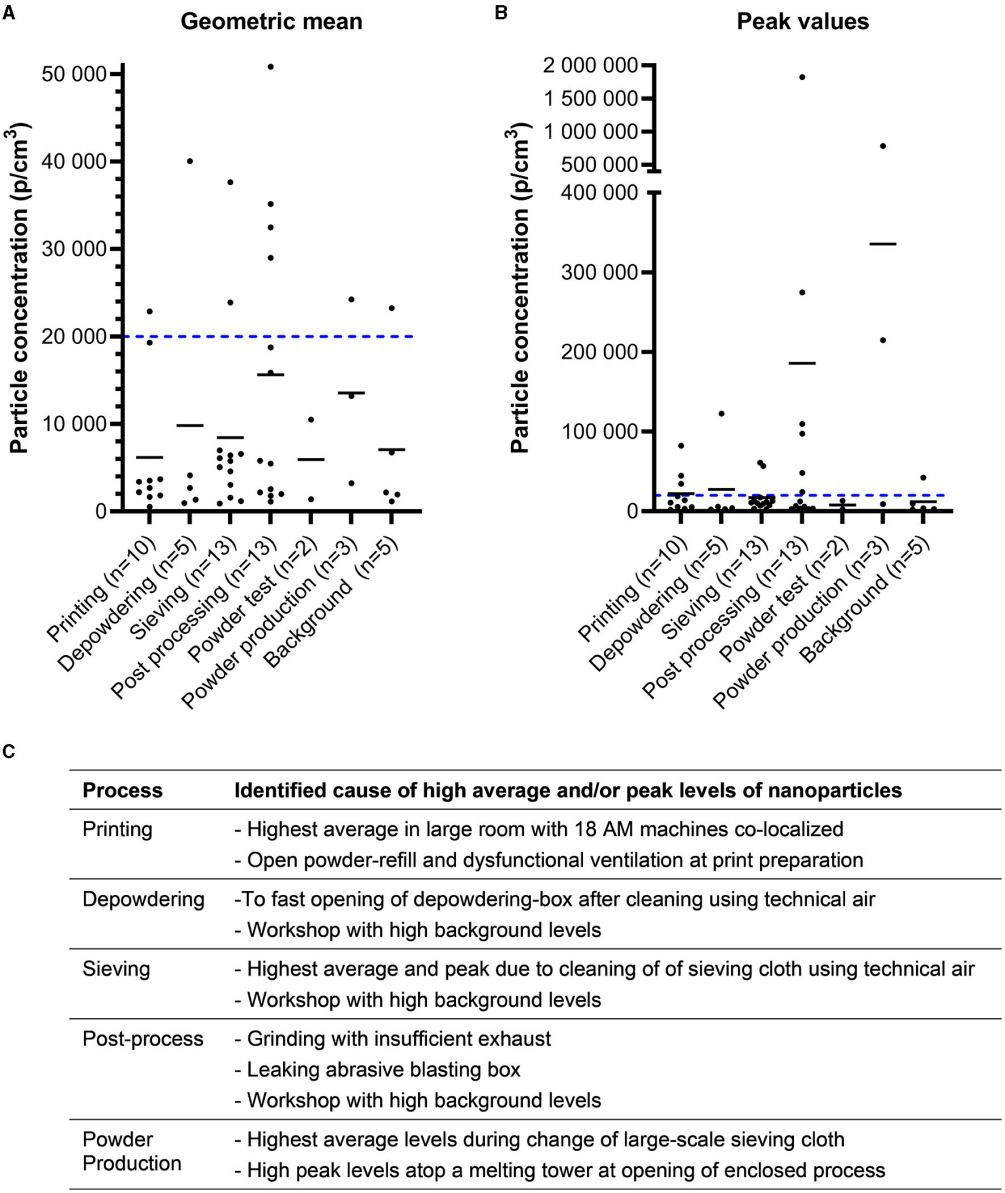
Nanoparticles. Concentration of 10–300 nanometer particles measured by Nanotracer. Values are presented as geometric mean **(A)** for the sampling period and the maximum value obtained **(B)**. The small lines in each group represent the arithmetic means. The blue dotted line represents a target value of 20,000 particles/cm^3^ suggested by the Finnish Institute of Occupational Health. Please note the different scaling of the *y*-axis on graph **(B)**. **(C)** Identified causes of high particle levels for different processes.

**Figure 4:**
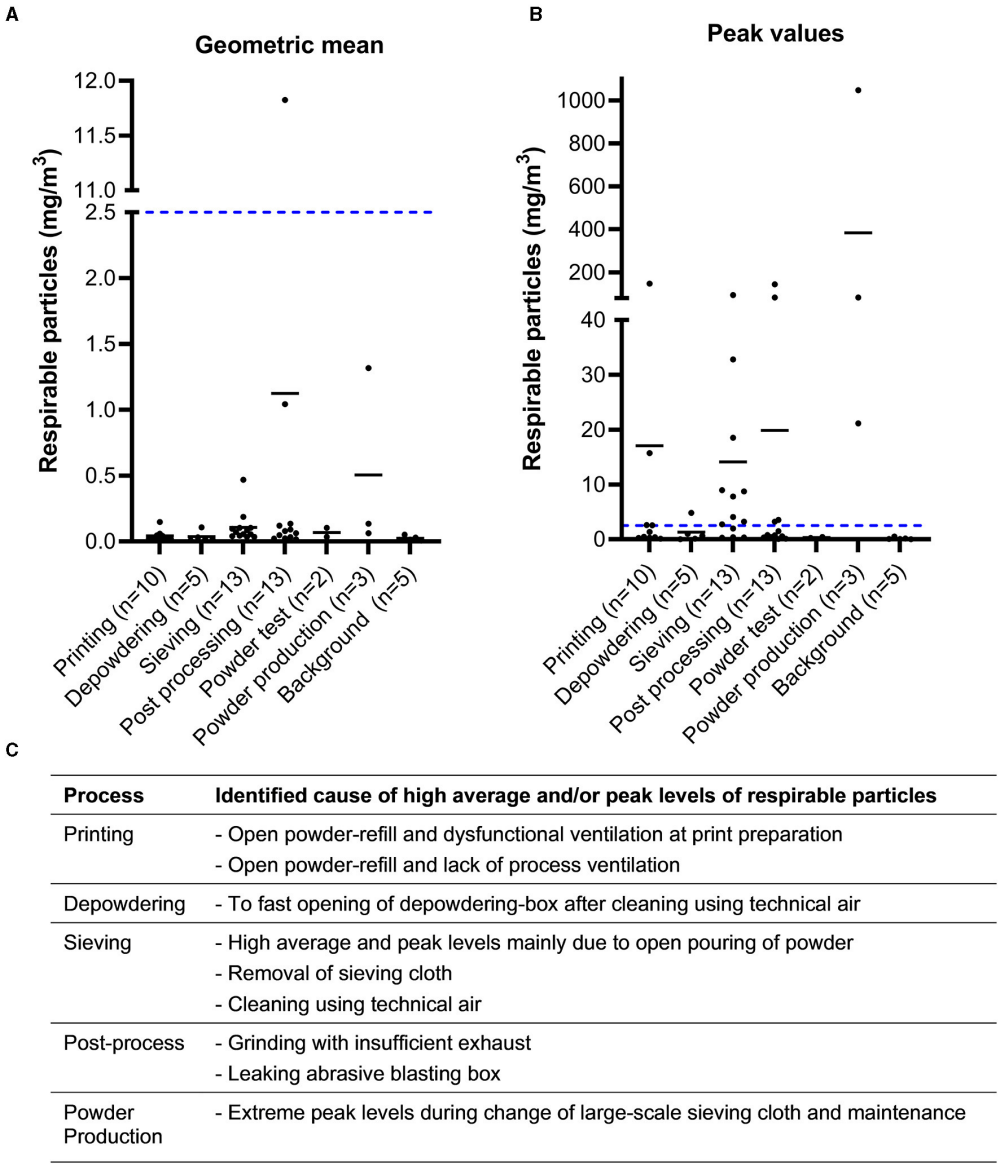
Respirable particles. Concentration of 0.3–10 μm sized particles as measured by Lighthouse. Values are presented as geometric mean **(A)** for the sampling period and the maximum value obtained **(B)**. The blue dotted line represents the Swedish OEL for respirable dust as comparison. Note that this is a limit for an 8 h average day, which must be determined through filter based sampling and gravimetric analysis to be legally binding. Please note the different scaling of the y-axis on graph B. **(C)** Identified causes of high particle levels for different processes.

Particle measurements were performed with two different types of instruments; Nanotracer was utilized for detecting nanoparticles (10–300 nm), Figure 3, while Lighthouse was used to measure respirable particles (0.3–10 μm), Figure 4. These direct-reading measurement techniques allow for a more detailed investigation of what type of process steps or equipment that entails potential exposure, Figures 3C, 4C. High particle levels were found as a result of inadequate work routines or use of technical air for cleaning, while some emissions resulted from faulty equipment or lack of suitable process ventilation. Also, large-scale AM production with several co-localized machines resulted in high background levels of nanoparticles. The powder production facility had high peaks in particles levels during maintenance and cleaning of a large-scale sieve and when opening the melting tower to refill materials for the next powder batch.

**Figure 5:**
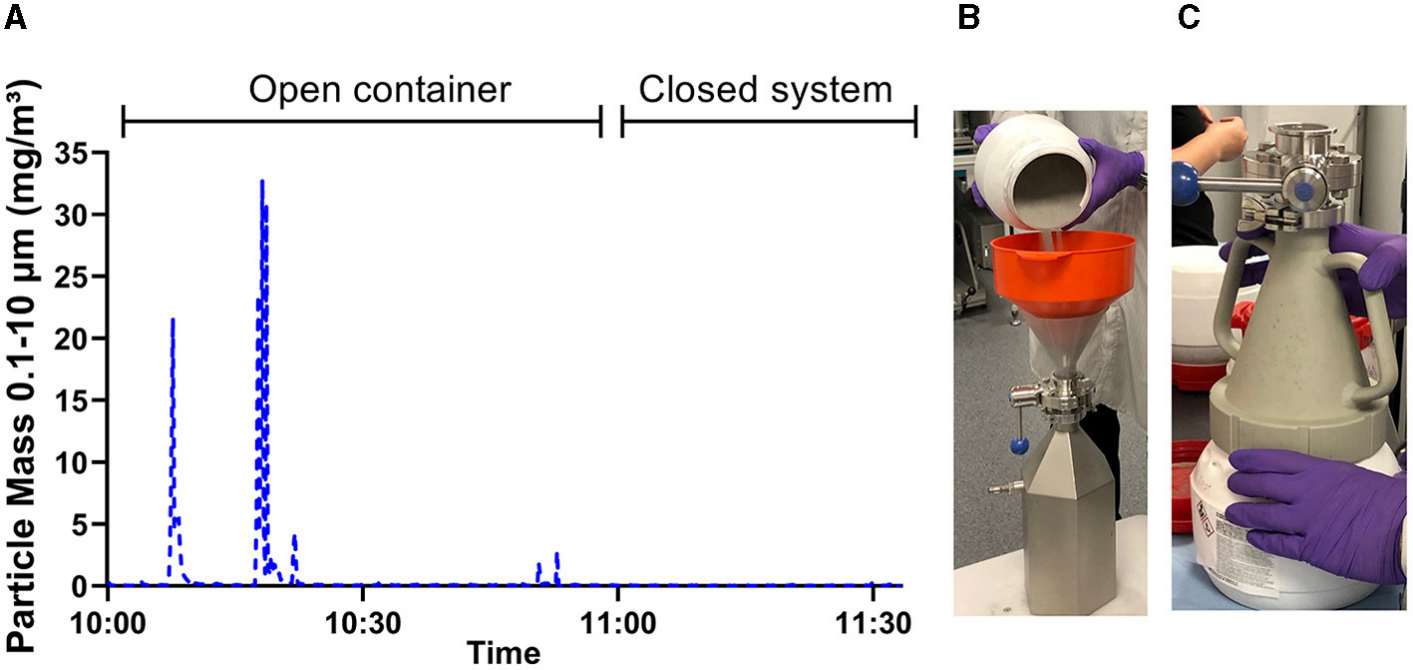
Use of particle measurement to verify preventive measures. **(A)** Estimated mass concentration of 0.3–10 μm sized particles as measured by Lighthouse and their variation over time. The graph clearly shows peak emissions of particles when powder is poured openly between containers **(B)**. When the task was repeated with a powder funnel to enclose the dusty work **(C)**, no change in particle levels were observed.

Direct-reading particle counters offers the possibility to quickly assess differences in particle emissions between various work routines and process steps. Figure 5 shows an example of how direct-reading instruments were used to evaluate the effectiveness of a closed system for powder refilling as a preventive measure against particle exposure.

### 3.2 Health examinations

In total, 48 AMMU participated in the study. These represented a clear majority of the exposed workforce within each company, where the average participation rate was 84% (range 67%−100%). Furthermore, 39 controls participated in the study, Table 4. The AMMU group were further analyzed as two subsets exposed to either titanium (Titanium, *n* = 16) or remaining metal alloys (Metal, *n* = 32). No differences in age, body mass index, or smoking status were present between exposed and controls. However, a significantly larger proportion of men were present in exposed groups as compared to the control group.

**Table 4:** Demographics.

	**Controls**	**AMMU**		
	**(*****n*** = **39)**	**All (*****n*** = **48)**	**Metal (*****n*** = **32)**	**Titanium (*****n*** = **16)**
Age (years)	40 (26–61)	34 (20–63)	34 (20–63)	33 (23–46)
Sex (men/women)	17/22	41/7^*^	26/6^*^	15/1^*^
Body mass index (kg/m^2^)	25 (19–35)	27 (19–44)	27 (19–44)	26 (20–32)
Smoker (yes/former/never)	0/5/34	4/3/41	3/2/27	1/1/14
Worked years (< 1/1–5/>5 years)	5/25/8	13/33/2	10/20/2	3/13/0
Physician-diagnosed asthma	2	3	2	1

^*^p < 0.001 chi-square.

#### 3.2.1 Questionnaire

**Figure 6:**
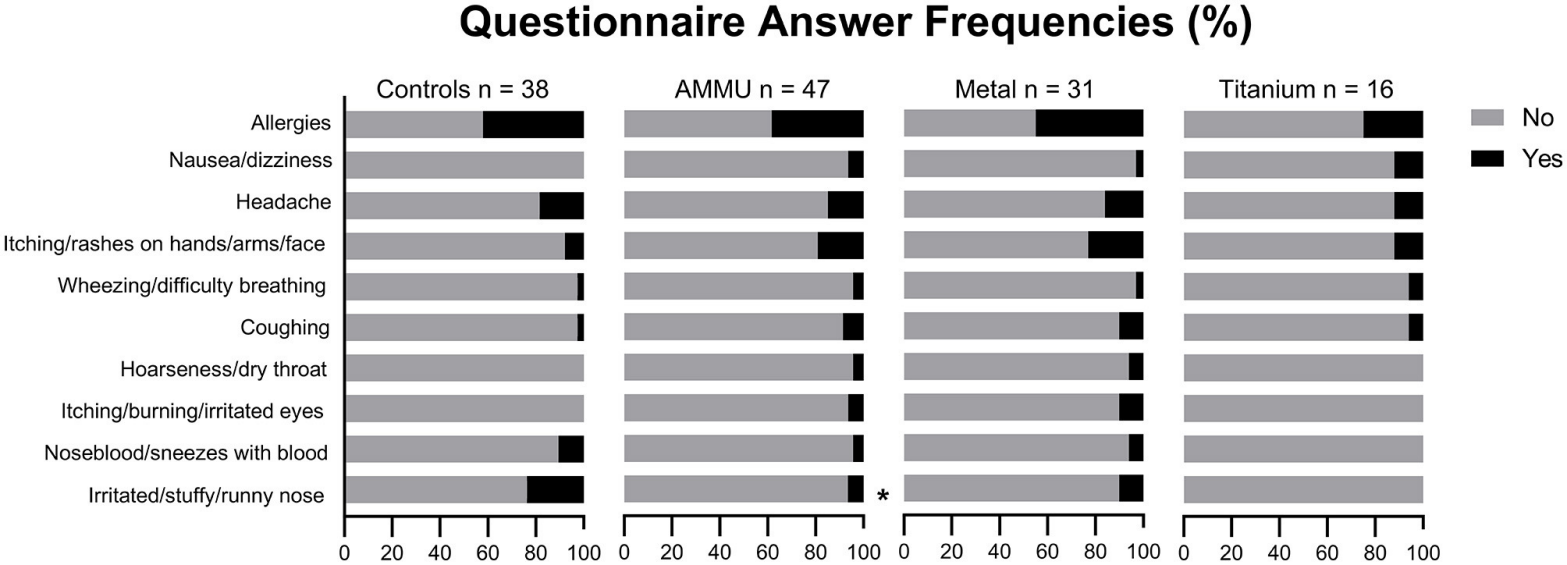
Answer frequencies in questionnaire. *p < 0.05 chi-square and Fishers exact test.

Figure 6 shows frequencies of self-reported symptoms that occur at least once a week. In total, 38 controls and 47 AMMU completed the questionnaire. One control and one AMMU (Metal) did not answer the questionnaire. Chi square and Fishers exact test showed that controls had more problems with irritated, stuffy, or runny noses than AMMU (*p* < 0.05) with an Odds Ratio (OR) of 4.4 (CI95% 1.1–17.9). However, when AMMU and controls were analyzed according to sex this was no longer the case. Both control women and AMMU women had a 29% frequency of irritated, stuffy, or runny noses (data not shown).

#### 3.2.2 Exposure markers

There are no Swedish limits for metals in urine, but the Finnish Institute of Occupational Health (FIOH) has used reference values for non-exposed individuals and biomonitoring action limits since the 1970s (52). Therefore, we use FIOH's values to interpret the investigated exposure markers. Urine samples were either analyzed for multiple metal elements or titanium depending on the powders used at the workshops. For multiple metal analyses, a total of 24 control and 30 AMMU remained after exclusion of urine samples, as described in methods, Table 5. On a group level, urinary metal levels in both AMMU and controls were below FIOH's limit for non-exposed individuals. However exposed individuals were identified, and some even exceeded FIOH's biomonitoring action limit.

**Table 5:** Exposure markers in controls and additive manufacturing machine users (AMMU).

**U-metals**		**Chromium**	**Cobalt**	**Iron**	**Manganese**	**Molybdenum**	**Nickel**
		**Specific gravity adjusted values (nmol/L)**					
**Ref. FIOH NE/BAL**		**10/20**	**25/130**	**–/–**	**10/–**	**1,340/–**	**50/100**
Controls total (*n* = 24)	Mon	16.4 (5.92–58.3)	4.35 (0.60–19.2)	636 (123–2150)	54.4 (1.76–575)	327 (51.4–1450)	36.8 (3.74–118)
	Fri	22.5 (5.29–175)	3.62 (0.03–24.9)	835 (71.4–2,800)	70.5 (8.12–217)	354 (3.00–1,640)	37.0 (8.67–147)
Controls men (*n* = 13)	Mon	17.0 (7.92–52.2)	4.15 (0.60–19.2)	664 (217–2,150)	41.7 (1.76–575)	357 (51.4–1,210)	41.2 (5.99–118)
	Fri	26.3 (9.38–175)	2.97 (0.05–6.54)	779 (71.4–1,760)	66.6 (8.12–166)	296 (3.00–1,640)	50.1 (16.5–147)
Controls women (*n* = 11)	Mon	15.7 (5.92–58.3)	4.63 (1.42–14.3)	604 (123–1,900)	74.5 (29.8–180)	294 (95.8–1,450)	31.8 (3.74–62.3)
	Fri	18.0 (5.29–46.4)	4.51 (0.03–24.9)	926 (202–2,800)	76.0 (30.0–217)	439 (217–1,020)	27.3 (8.67–76.3)
AMMU total (*n* = 30)	Mon	14.7 (4.78–71.0)	4.34 (0.61–26.4)	472 (133–3,370)	50.7 (3.84–225)	437 (63.5–2,310)	27.3 (6.79–190)^*^
	Fri	16.8 (3.91–103)	4.79 (1.12–139)	499 (105–1,180)^*^	58.6 (4.69–421)	509 (117–3,920)	33.5 (7.77–198)
AMMU men (*n* = 25)	Mon	14.5 (4.78–71.0)	3.46 (0.61–23.5)	411 (133–1,220)^*^	51.1 (3.84–225)	438 (63.5–2,310)	26.7 (6.79–190)^*^
	Fri	14.5 (3.91–35.7)	3.59 (1.27–10.4)	430 (105–1,160)^*^	51.0 (4.69–188)	518 (117–3,920)	30.4 (7.77–92.8)
AMMU women (*n* = 5)	Mon	16.2 (8.42–35.5)	16.9 (8.96–26.4)^*^	1,300 (712–3,370)	48.9 (24.7–95.5)	434 (207–1,370)	30.9 (11.4–60.3)
	Fri	31.0 (14.2–103)	20.3 (1.12–139)	874 (645–1,180)	118 (49.1–421)	466 (186–786)	54.6 (11.6–198)

Values are geometric mean (minimum–maximum). The bold values are indicate the limit/reference values stated in the left-most column of the tables.

FIOH, Finnish Institute of Occupational Health; NE, Reference limit for non-exposed; BAL, biomonitoring action limit.

^*^Statistically significant difference between AM and controls same weekday according to Mann–Whitney U-test p < 0.05.

Titanium samples from 14 controls and 14 AMMU remained after exclusion of urine samples, as described in methods. All except three samples were below the report limit of 20 nmol/L. Two controls had detectable titanium levels, but these were well below FIOH's limit for non-exposed individuals. One titanium-exposed AMMU had a level of 720 nmol/L in the Friday sample, which is above FIOH's non-exposed-limit of 680 nmol/L.

#### 3.2.3 Clinical markers

**Figure 7:**
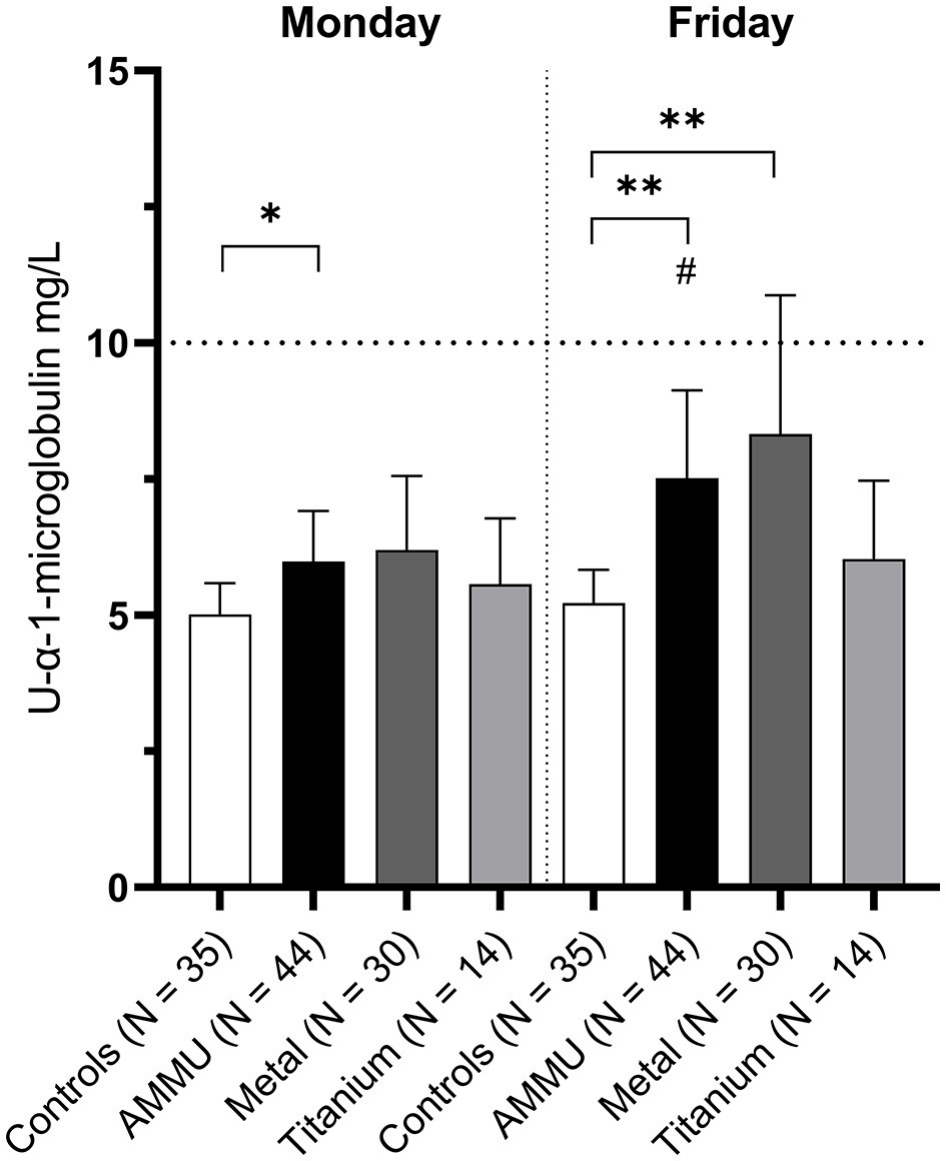
Alpha-1-microglobulin in urine. Values are geometric mean. The dotted line indicates the reference values for both men and women (10 mg/L). ^*^/^**^ = *p* < 0.05/0.01 compared to control same weekday, Mann–Whitney *U*-test. ^#^*p* < 0.05 Friday vs Monday in respective group, Wilcoxon signed rank test.

Urinary levels of α-1-M were significantly higher in the AMMU group compared to controls, in both Monday and Friday samples, Figure 7. Stratifying for type of exposure, the Metal group had higher levels α-1-M on Fridays compared to controls. There was an increase in α-1-M within the AMMU group when comparing Friday samples to Monday samples. In sex-specific analysis, α-1-M levels in AMMU men were significantly higher on both Monday and Friday compared to control men, and levels increased during the workweek, Supplementary Table 1. However, α-1-M levels were within clinical reference values for all groups on a group level.

Clinical markers in plasma were also within clinical reference values in all groups, Supplementary Table 2. Although significant differences were found between exposed and controls. ASAT was higher in the AMMU group, and specifically the Metal group in stratified analyses, compared to the control group. The AMMU group had lower ApoA-1 compared to controls. However, sex-specific statistical analysis of ASAT and ApoA-1 did not reveal any differences between groups.

Stratified analyses for titanium-exposed operators showed no significant differences in clinical markers in neither urine nor plasma compared to controls.

#### 3.2.4 Lung/airway tests

Spirometry results, expressed as percentage of predicted values using the Hedenström references (44, 45), are shown in Table 6. There were no statistically significant differences between AMMU and controls and the geometric mean of the groups were within reference values.

**Table 6:** Spirometry results in percentage of predicted values.

	**Controls**	**AMMU**		
	**(*****n*** = **39)**	**All (*****n*** = **47)**	**Metal (*****n*** = **31)**	**Titanium (*****n*** = **16)**
**Spirometry**				
FEV1/FVC (% pred.)	79 (66–93)	80 (66–94)	79 (66–94)	81 (72–88)
FEV1 (% pred.)	88 (62–122)	93 (74–113)	92 (75–113)	93 (74–107)
FVC (% pred.)	89 (67–118)	92 (71–107)	92 (75–107)	91 (71–104)

Values are geometric mean (minimum–maximum).

FEV1, forced expiratory volume in one second; FVC, forced vital capacity.

Reference values for healthy individuals are 80%−120%.

A subset of study participants was further subjected to more in-depth lung function tests: FeNO, IOS, and breath analyses by PEx, Table 7. AMMU had significantly higher FeNO levels than controls (*p* < 0.05). When stratifying the analyses, only Titanium exposed had significantly higher FeNO levels. All controls were within normal range (< 25 ppb). Among the exposed individuals, some showed high FeNO levels, such as one titanium-exposed man with a FeNO of 51 ppb. No statistically significant differences in IOS parameters were found between groups. However, there were individuals with results outside normal range in the exposed group. PEx samples showed no statistically significant differences regarding number of particles per breath, SP-A levels, albumin, or PC lipids. PC lipids are displayed in Supplementary Table 3.

**Table 7:** Results of the lung function tests FeNO and IOS along with breath analyses.

	**Controls**	**AMMU**		
	**(*****n*** = **25)**	**All (*****n*** = **29)**	**Metal (*****n*** = **14)**	**Titanium (*****n*** = **15)**
**Fraction of exhaled NO**				
FeNO (ppb)	13 (5–25)	17 (5–51)^*^	14 (5–38)	20 (15–51)^*^
**Impulse oscillometry**				
IOS R5 Hz (*z*-score)	−0.24 (−2.14–3.43)	−0.40 (−1.16–2.06)	−0.62 (−1.86–1.68)	−0.19 (−1.71–2.06)
IOS R5–20 Hz (*z*-score)	−0.24 (−1.48–1.78)	−0.35 (−1.48–2.28)	−0.41 (−1.48–2.28)	−0.28 (−1.16–1.21)
IOS AX (*z*-score)	0.50 (−0.78–4.97)	0.05 (−0.94–6.07)	0.05 (−0.94–6.07)	0.04 (−0.77–1.59)
**Particles in exhaled air**				
PEx Number/breath	35,700 (7,400–122,100)	26,900 (2,300–97,100)	18,700 (2,300–64,900)	37,600 (7,400–97,100)
PEx SP-A (%PEx)	3 (2–4)	3 (2–6)	3 (2–5)	3 (2–6)
PEx Albumin (%PEx)	4 (3–13)	4 (2–7)	3 (2–5)	4 (3–7)
PEx Albumin/SP-A	1.36 (0.75–6.16)	1.23 (0.57–2.59)	1.11 (0.57–2.36)	1.34 (0.58–2.59)

Values are geometric mean (minimum–maximum) for FeNO and PEx, and IOS z-scores are presented as median (minimum–maximum).

^*^p < 0.05 compared to control, Mann–Whitney U-test.

FeNO, fraction of exhaled NO; IOS, impulse oscillometry; AX, area of reactance; R5/20, resistance at 5/20 Hz; PEx, particles in exhaled air; SP-A, surfactant protein A.

Impulse oscillometry z-scores calculated according to Kjellberg et al. (47).

## 4 Discussion

In this study we have had access to several metal AM companies in Sweden using various techniques and powders. We have mapped occupational exposures along the whole process chain and have delved into possible risks associated with metal AM. Our measurements confirm that, even though inhalable dust levels are generally low compared to Swedish OELs, specific work tasks can emit high levels of airborne particles, such as open powder handling or post-processing of builds, risking possible exposure. This is in line with our earlier studies on a single site that had potential exposure, which were reduced after the company introduced several preventive actions (5, 32, 36). Expanding our investigations by increasing the number of companies and AM processes, we confirm that the chosen methodology can be successfully applied in the AM setting. Our findings highlight the importance of continued evaluation of occupational safety to ensure the effectiveness and relevance of preventive measures, including the importance of continued education of personnel regarding safety and work routines. Moreover, we found that large-scale AM production can lead to an increased risk of exposure due to the increased amounts of powder, time spent with dusty work tasks, and co-localization of several AM machines with concurrent activities. It is essential to recognize that initial studies of smaller AM facilities with low exposure results can lead to a false sense of security when performing risk assessment for large-scale productions based such results. Regarding health effects, we found signs of possible effects on renal function and indications of airway inflammation.

### 4.1 Exposure measurements

#### 4.1.1 Dust and metals in air

The AM facilities had in general low levels of inhalable dust and metals compared to the current OELs in Sweden, and the exposure of AMMU working with LB-PBF, EB-PBF, or BJT methods were all well below OELs. This is in line with our earlier studies (5) as well as other studies in metal AM (6, 53).

Different metals have varying effect on health, which form the basis for their OELs. However, Swedish OELs are pragmatic, and exposure below OELs does not guarantee the absence of adverse health effects in all workers. It is important to note that the Swedish OEL for cobalt (0.02 mg/m^3^) is 10 to 100-fold lower than that of the other investigated metals, and thus often reaches its OEL first when alloys containing cobalt is used. The majority of the air measurements herein had more chromium and nickel than cobalt due to the materials used. Recent studies have highlighted the need for lower nickel OEL to ensure worker safety and health (54).

#### 4.1.2 Particle measurements

Throughout the whole AM chain, several processes contribute to the AMMU's daily exposure, and each preventive measure helps lower the total exposure. We used particle counting instruments in parallel with collection of inhalable dust for gravimetric and metal analysis to identify specific work tasks with increased exposure risk.

The investigated AM machines and the printing process itself did not emit nano- and respirable particles because these were enclosed systems, as were most depowdering stations and sieves. Emission levels did not vary between AM techniques but rather it depended on the equipment and routines used in the manual steps before and after printing. High peaks in particle levels were associated by open powder handling, maintenance/cleaning of machines, and insufficient or lack of exhaust ventilation. Co-localization of several AM machines in one facility resulted in high general levels of nanoparticles. Post processes and powder production had prominent particle emissions compared to the AM workshops. Interestingly, post processes tend to cause high nanoparticle emissions, as does the melting process in powder production, whereas respirable particles dominated during maintenance of large-scale sieving at a powder production facility. The techniques used for post processing of the builds are not AM-specific, i.e., grinding, sawing, milling. However, some of the new metal alloys used in AM may pose new risks when using these traditional metal working techniques. Thus, the whole AM value chain must be evaluated to ensure proper occupational hygiene as the industry continues to grow.

Since these particle sizes are not visible to the naked eye nor do they notably contribute to the mass when collecting dust on filters, particle counting instruments are valuable tools to identify emission sources. During the project, all companies received measurement reports with suggestions for preventive measures. Herein, particle measurements proved to be very pedagogic as it was possible to show peak emissions during specific activities and it helped to prioritize areas for preventive measures. Figure 5 shows an example where particle counters clearly identified high peaks of respirable particles during open powder pouring between containers, leading the company to introduce a powder funnel to enclose the powder handling, which proved to be effective.

### 4.2 Health examinations

#### 4.2.1 Questionnaires

Questionnaires with self-reported symptoms are often used to investigate possible exposure-related health effects (55, 56) and may be a valuable tool while investigating new and upcoming industries. Here, questionnaires were utilized to investigate self-reported symptoms related to indoor air exposure (42), alongside the occurrence of asthma and allergies, Figure 6. Interestingly, a significantly lower proportion of AMMU reported problems with irritated, stuffy, or runny noses compared to the controls who worked in office environments. Problems in the upper airways among office workers have been reported in previous studies (57). Interestingly, women tend to report these kinds of health symptoms more frequently than men (58). Therefore, the differences between AMMU and controls may be skewed by the larger proportion of women in the control group. In fact, the women in the AMMU group reported the same frequency of problems with irritated, stuffy, or runny noses as the control women. The reason for higher prevalence of self-reported symptoms among women are widely discussed and consensus seems to be that women have increased sensitivity in general (59, 60).

#### 4.2.2 Exposure markers

The biological burden of metals can be assessed through analysis of urinary metals, which can be a valuable tool if reference or action levels for the specific elements are available (20–23). Here, we analyzed metals in urine and compared them to reference values for non-exposed individuals and to biomonitoring action limits proposed by FIOH (52).

Overall, the exposure to metals was low and well below reference values for non-exposed on a group level for most metals in both Monday and Friday samples. This differs from our previous studies of metal AM workers, where we saw tendencies that urinary metal levels coincided with levels of airborne metals and increased over a workweek (5). The lower exposure can be explained by improved work environment as well as work routines for companies participating in the previous study. Moreover, several new companies were included in the current study, most of which were small-scale AM facilities with good work practice. Despite this, we still see individuals exceeding the biomonitoring action limits for nickel and cobalt proposed by FIOH. Moreover, some individuals had increased metal levels over the workweek. As always, there will be individual variation in urinary metal levels (61). Upon closer examination of the individuals with higher urinary metal levels, we observed associations of the exceeding levels with high workload, inadequate respiratory protection, or changes in the work environment, such as increased production, more AM machines, or the hiring of new staff with the possibility of improper work routine introductions. This underlines the value of continuously monitoring exposure through urine analyses.

However, there is an urgent need for national reference values, due to variations in general urinary metal levels between countries (61, 62). Cobalt levels were significantly higher in AMMU women than control women in Monday samples with a similar trend in Friday samples, yet still below FIOH's limit for non-exposed individuals. One AMMU woman even exceeded the FIOH biomonitoring action limit for cobalt at the end of a workweek. Iron-deficiency has a higher prevalence in women and this leads to an increased uptake of cobalt (63), which can present itself as a higher cobalt excretion (61). This may explain our findings with higher cobalt in women on a group level and suggests a need for sex-specific risk assessment for metal exposure.

#### 4.2.3 Clinical markers

Various organs, such as kidney, liver, and heart, can become secondary targets for inhaled or ingested particles. We have chosen existing clinical markers for vascular, renal, and hepatic function that has previously been used (5) to survey possible effects on these organs.

We observed significant differences between AMMU and controls for the marker for renal function (α-1-M). Interestingly, men in the AMMU and Metal group had significantly higher α-1-M levels in Friday samples compared to control men, as well as significantly increased α-1-M levels from Monday to Friday, Supplementary Table 1. Similar effects on biomarkers for renal function have been observed, even at low exposure to metals (32, 64). However, in women, no apparent difference between AMMU and controls were present that may indicate a sex-specific difference. Curiously, when the AMMU group was divided into Titanium and Metal exposed, α-1-M was significantly altered in the Metal group but not in the Titanium group. This finding seems consistent with the extremely low or undetectable levels of titanium in the urine of AMMU individuals, with only one individual working with powder production who had a titanium level just above FIOH's reference value for non-exposed individuals.

Historically, titanium has had a reputation of being inert and thus biocompatible, leading to respiratory protection being seldomly used when working with titanium powders in the AM industry. However, recent studies implies potential hazards of titanium exposure (65). In the present study, we cannot conclude whether titanium powder is taken up by the lung or if it cannot reach the circulation and, subsequently, the urine. This must be further studied since accumulation of titanium within the lung may have other unwanted effects.

The statistical differences in ASAT and ApoA-I appears to be caused by the different sex distribution between exposed and controls. Women tend to have lower ASAT and higher ApoA-I than men (66, 67), which is also reflected in the reference interval for these clinical markers. Indeed, when men and women were analyzed separately, ASAT and ApoA-I were not significantly altered between controls and AMMU.

In summary, the clinical analyses reveal an interesting observation: the marker for renal function is significantly affected already at these low urinary metal levels indicating that this is probably a suitable biomarker for the metals used in the present study.

#### 4.2.4 Lung/airway tests

Neither spirometry nor IOS showed any differences between exposed and controls at a group level. Most AMMU wore Powered Air Purifying Respirators during work with powders containing nickel and cobalt decreasing individual exposure. It should be noted that only two of the AMMU participants have worked more than 5 years, while one third of the participants had < 1-year experience with metal AM, and the remaining between 1 and 5 years. Thus, it may be too soon to see any chronic negative effects on the respiratory system.

Interestingly, FeNO were significantly higher in the Titanium group than in controls indicating airway inflammation. The Titanium workers did not wear respiratory protection because the material is traditionally considered inert. Our result may suggest airway irritation due to inhalation of titanium particles, which should be studied further in the future.

The composition and function of the lung surfactant can be altered by direct interactions with inhaled nanoparticles as well as by inflammatory processes (68–71). Thus, lung surfactant phospholipids and proteins may serve as potential biomarkers of early adverse changes and airway disease, particularly in the small airways like asthma and chronic obstructive pulmonary disease. In a previous study, including operators in polymer AM industry, we found significant differences in the composition of saturated and unsaturated PC lipids suggesting an exposure-related effect that may be related to an inflammatory process in the small airways (17). Here, we found no changes in the PC lipid pattern nor in the levels of SP-A and albumin between groups. The fact that differences were observed in FeNO but not in PEx suggests that inflammation could be located in the upper airways in the Titanium group rather than in the small airways.

#### 4.2.5 Limitations and future perspectives

The main limitation is the relatively low number of study participants and the variability in the work tasks they perform, with varying frequencies. Several of the companies were start-ups or research and process development facilities with few AM operators, whom also had a lot of office time. This resulted in small groups of workers from each company, and many of them had low and/or infrequent exposure. Moreover, most of the workers were new in the AM industry. This makes any conclusions regarding health effects uncertain. More studies on a larger, more frequently exposed groups are needed. Future studies should also investigate respirable dust and metal levels to further clarify the relationship between health effects and exposure to fine dust. It is important to continue to monitor this industry as it grows. Our study gives valuable insights to exposure risks in this new industry and provides a foundation for future investigations. It should be noted that our study only focused on dust, metals, and particles, whereas other risks such as solvents, binders, and noise among other also occur in these work environments. These potential other exposures may have influenced our results. Measurements of other exposures beyond the ones studied herein, especially the liquid bonding agent used in BJT, should be included in future studies regarding the overall health effects in AM environments.

## 5 Conclusion

The study shows the success of the chosen methodological approach in studying exposure risks in AM environments and potential health risks. Based on current OELs for airborne dust and metals, the exposure is relatively low apart from specific work tasks with high particle emissions. However, interpretation of exposure-related risk should be performed with caution since AM involves several metals that may have negative effects on health. Despite only a few cases showing metal levels in urine exceeding Finnish biomonitoring action limits, the study revealed kidney function could be affected in the AM operators compared to controls. Moreover, inhalation of titanium may cause airway inflammation. Since all but two participants have been working < 5 years, long-term studies of AM operators' health are needed before we can accept or reject the observed effects on health.

## Data availability statement

The raw data supporting the conclusions of this article will be made available by the authors, without undue reservation.

## Ethics statement

The studies involving humans were approved by Swedish Ethical Review Authority (Dnr 2016/112-31, 2019-03529, and 2019-03536). The studies were conducted in accordance with the local legislation and institutional requirements. The participants provided their written informed consent to participate in this study.

## Author contributions

MA: Conceptualization, Data curation, Formal analysis, Investigation, Methodology, Project administration, Resources, Validation, Visualization, Writing – original draft, Writing – review & editing. A-CA: Conceptualization, Data curation, Investigation, Methodology, Project administration, Resources, Supervision, Validation, Writing – review & editing. SK: Investigation, Writing – review & editing. SL: Conceptualization, Data curation, Formal analysis, Investigation, Methodology, Resources, Supervision, Validation, Visualization, Writing – review & editing. AO: Conceptualization, Resources, Supervision, Writing – review & editing. HK: Conceptualization, Funding acquisition, Investigation, Methodology, Project administration, Supervision, Validation, Writing – review & editing.
