# Supplementary material for: Occupational exposure and health surveys at metal additive manufacturing facilities
Source: Front Public Health. 2023 Nov 20;11:1292420. doi: 10.3389/fpubh.2023.1292420 (PMC10694287; doi:10.3389/fpubh.2023.1292420)
Supplement: Supplementary file 1 [file Data_Sheet_1.PDF]

Supplementary Material

1 Supplementary Experimental Procedures, Questionnaire

Participant details

Birth year ………………………...……………………………………………………..

Name ……………..……………..…………………………………………………………

Workplace…………………….……………………………………………………………

Department………..………….…………………………………………………………….

Has been working here since (year, month) …...…………………………………………..

Sex: Man ☐ Woman ☐
Date (YYYY/MM/DD) ………………………….………………………………………..

Current discomforts

<u>Underline</u> what symptoms you have, check with yes / no boxes to indicate if you have any problems, and at which workplace you have symptoms, and when the symptoms began.

	Yes	No	Which work tasks do / did you have?	Symptoms since (enter year, month)
1. Do you have irritated, stuffy or runny nose on an almost daily basis?	☐	☐	………………………	……………
2. Do you have nosebleeds or sneezes mixed with blood on an almost daily basis?	☐	☐	……………………	……………
3. Do you have itching, burning or irritated eyes on an almost daily basis?	☐	☐	………………………	……………
4. Do you have hoarseness or dry throat on an almost daily basis?	☐	☐	………………………	……………
5. Do you cough on an almost daily basis?	☐	☐	………………………	……………
6. Wheezing in the chest or difficulty breathing at least once a week?	☐	☐	……………………….	……………
7. Have you been diagnosed with asthma by a doctor?	☐	☐	……………………….	……………
8. Do you have itching or rashes on hands, arms, or face?	☐	☐	……………………….	……………
9. Do you have headache at least one day per week?	☐	☐	……………………….	……………
10. Do you have nausea or dizziness at least one day per week?	☐	☐	……………………….	……………

11. If you have any of the discomforts in question 1-10, what do you think it is due to?			…………………………………………………… ……………………………………………………
	Yes	No	
12. Do you have any allergies?	☐	☐	If yes,<br>Please state when your allergy started…………….<br>What are you allergic to?.......................................<br>What period of the year do you have problems with your allergy?<br>From………………… Until…………………….
13. Are you a smoker?	☐	☐	If yes,<br>How much do you smoke?<br>…………..cigarettes per day
14. Are you a former smoker?	☐	☐	If yes,<br>When did you stop smoking??<br>Year……………………………

2 Supplementary Figures and Tables

Table 1 Clinical marker in urine: alpha-1-microglobulin (α-1-M). Reference value for men and women < 10 mg/L.

	**Controls**<br>Total n = 35<br>Men n = 17<br>Women n = 18	**AMMU**<br>Total n = 44<br>Men n = 38<br>Women n = 6	**Metal**<br>Total n = 30<br>Men n = 25<br>Women n = 5	**Titanium**<br>Total n = 14<br>Men n = 13<br>Women n = 1
		**α-1-M mg/L Monday**		
**All**	5.01 (4.49 - 18.30)	5.99 (4.49 - 28.20)*	6.20 (4.49 - 28.20)	5.57 (4.49 - 12.20)
**Men**	5.14 (4.49 - 18.30)	6.14 (4.49 - 28.20)	6.52 (4.49 - 28.20)	5.51 (4.49 - 12.20)
**Women**	4.89 (4.49 - 7.54)	5.12 (4.49 - 6.83)	4.88 (4.49 - 6.83)	6.47
		**α-1-M mg/L Friday**		
**All**	5.22 (4.49 - 19.30)	7.52 (4.49 - 46.50)**#	8.33 (4.49 - 46.50)**	6.03 (4.49 - 10.80)
**Men**	4.76 (4.49 - 8.26)	7.98 (4.49 - 46.50)**#	9.12 (4.49 - 46.50)**	6.17 (4.49 - 10.80)
**Women**	5.70 (4.49 - 19.30)	5.14 (4.49 - 6.90)	5.28 (4.49 - 6.90)	4.49
		**α-1-M/crea mg/mM Monday**		
**All**	0.42 (0.13 - 1.59)	0.50 (0.17 - 2.39)	0.57 (0.17 - 2.39)	0.39 (0.23 - 1.08)
**Men**	0.40 (0.13 - 1.59)	0.50 (0.22 - 2.39)	0.58 (0.22 - 2.39)	0.39 (0.23 - 1.08)
**Women**	0.44 (0.19 - 1.14)	0.51 (0.17 - 1.52)	0.54 (0.17 - 1.52)	0.38
		**α-1-M/crea mg/mM Monday**		
**All**	0.51 (0.14 - 2.21)	0.59 (0.18 - 1.91)	0.64 (0.21 - 1.91)	0.49 (0.18 - 1.18)
**Men**	0.47 (0.23 - 2.21)	0.58 (0.18 - 1.91)	0.64 (0.21 - 1.91)	0.49 (0.18 - 01.18)
**Women**	0.55 (0.14 - 2.19)	0.60 (0.36 - 1.12)	0.64 (0.36 - 1.12)	0.44

Values are geometric mean (minimum – maximum).
*/** = p<0.05/0.01 compared to control same weekday, Mann Whitney U-test.
# = p<0.05 Friday vs Monday in respective group, Wilcoxon signed rank test.
Reference value were obtained from the Clinical Chemistry section of Linköping University Hospital at the time of analysis.

Table 2 Clinical markers in plasma.

Clinical marker in plasma	Ref.	Controls<br>Total n = 39<br>Men n = 17<br>Women n = 22	AMMU<br>Total n = 48<br>Men n = 41<br>Women n = 7	Metal<br>Total n = 32<br>Men n = 26<br>Women n = 6	Titanium<br>Total n = 16<br>Men n = 15<br>Women n = 1
**ASAT (µkat/L)**					
All		0.43 (0.25 - 1.06)	0.50 (0.27 - 3.1)*	0.53 (0.27 - 3.1)*	0.45 (0.28 - 0.8)
Men	< 0.76	0.52 (0.32 - 1.06)	0.50 (0.28 - 3.1)	0.52 (0.31 - 3.1)	0.47 (0.28 - 0.8)
Women	< 0.61	0.38 (0.25 - 0.57)	0.51 (0.27 - 1.58)	0.56 (0.27 - 1.58)	0.28
**ALAT (µkat/L)**					
All		0.29 (0.11 - 0.61)	0.34 (0.14 - 1.09)	0.33 (0.14 - 1.09)	0.34 (0.16 - 0.64)
Men	< 1.2	0.33 (0.15 - 0.61)	0.36 (0.17 - 1.09)	0.35 (0.21 - 1.09)	0.36 (0.17 - 0.64)
Women	< 0.76	0.26 (0.11 - 0.58)	0.24 (0.14 - 0.48)	0.26 (0.14 - 0.48)	0.16
**ALP (µkat/L)**					
All	0.7 - 1.9	0.90 (0.32 - 1.49)	0.97 (0.56 - 1.83)	0.94 (0.58 - 1.83)	1.06 (0.56 - 1.79)
Men		0.87 (0.32 - 1.41)	1.01 (0.6 - 1.83)	0.96 (0.6 - 1.83)	1.10 (0.79 - 1.79)
Women		0.93 (0.48 - 1.49)	0.80 (0.56 - 1.32)	0.85 (0.58 - 1.32)	0.56
**ApoA1 (g/L)**					
All		1.54 (1.07 - 2.19)	1.43 (1.03- 2.32)*	1.44 (1.03 - 2.19)	1.43 (1.15 - 2.32)
Men	1-1.8	1.46 (1.07 - 1.82)	1.41 (1.03 - 2.19)	1.42 (1.03 - 2.19)	1.38 (1.15 - 1.73)
Women	1.1-2.1	1.60 (1.26 - 2.19)	1.61 (1.25 - 2.32)	1.51 (1.25 - 1.79)	2.32
**ApoB (g/L)**					
All	0.6-2	0.95 (0.58 - 1.78)	0.91 (0.54 - 1.64)	0.90 (0.54 - 1.64)	0.94 (0.57 - 1.45)
Men		0.97 (0.69 - 1.37)	0.92 (0.54 - 1.64)	0.90 (0.54 - 1.64)	0.94 (0.57 - 1.45)
Women		0.94 (0.58 - 1.78)	0.89 (0.57 - 1.09)	0.89 (0.57 - 1.09)	0.87
**ApoB/ApoA-I**					
All		0.62 (0.33 - 1.41)	0.64 (0.33 - 1.21)	0.63 (0.33 - 1.21)	0.66 (0.38 - 1.18)
Men	<0.9	0.66 (0.38 - 1.04)	0.65 (0.36 - 1.21)	0.64 (0.36 - 1.21)	0.68 (0.41 - 1.18)
Women	<0.8	0.58 (0.33 - 1.41)	0.55 (0.33 - 0.74)	0.59 (0.33 - 0.74)	0.38
**SAA1/PON1 (ng/U)**					
All		13.4 (2.01 - 127)	14.0 (2.56 - 459)	14.7 (2.67 - 459)	12.6 (2.56 - 81.0)
Men	-	9.46 (2.01 - 31.5)	13.2 (2.56 - 459)	14.0 (2.67 - 459)	12.0 (2.56 - 81.0)
Women	-	17.4 (3.89 - 127)	20.2 (5.3 - 53.3)	19.07 (5.3 - 53.3)	26.93

Values are geometric mean (minimum – maximum).
* = p<0.05 compared to controls, Mann Whitney U-test.
Reference values were obtained from the Clinical Chemistry section of Linköping University Hospital at the time of analysis.
ASAT: aspartate aminotransferase; ALAT: alanine aminotransferase; ALP: alkaline phosphatase; ApoA-I: apolipoprotein A-I; ApoB: apolipoprotein B; SAA1: serum amyloid A; PON1: paraoxonase/arylesterase.

Table 3 Phosphatidylcholine (PC) lipids in exhaled particles.

	Controls<br>n = 24	AMMU<br>n = 29	Metal<br>n = 14	Titanium<br>n = 15
**PC 14:0_14:0**	0.003 (0.001-0.007)	0.003 (0.001-0.008)	0.003 (0.002-0.004)	0.004 (0.001-0.008)
**PC 14:0_16:0**	0.082 (0.061-0.116)	0.084 (0.050-0.118)	0.083 (0.061-0.101)	0.084 (0.050-0.118)
**PC 15:0_16:0**	0.017 (0.012-0.024)	0.017 (0.012-0.025)	0.017 (0.013-0.025)	0.016 (0.012-0.022)
**PC O-16:0_16:0**	0.002 (0.001-0.003)	0.002 (0.002-0.003)	0.002 (0.002-0.003)	0.002 (0.002-0.003)
**PC 16:0_16:1**	0.090 (0.068-0.125)	0.086 (0.063-0.127)	0.087 (0.063-0.116)	0.085 (0.065-0.127)
**PC 14:0_18:1**	0.007 (0.004-0.018)	0.007 (0.005-0.013)	0.007 (0.005-0.010)	0.007 (0.005-0.013)
**PC 14:0_18:0**	0.002 (0.001-0.003)	0.002 (0.001-0.003)	0.002 (0.001-0.003)	0.002 (0.001-0.003)
**PC 16:0_16:0**	0.586 (0.414-0.651)	0.591 (0.501-0.671)	0.584 (0.501-0.671)	0.599 (0.527-0.653)
**PC 16:0_17:1**	0.004 (0.003-0.006)	0.004 (0.003-0.008)	0.004 (0.003-0.008)	0.004 (0.003-0.005)
**PC 16:0_17:0**	0.012 (0.007-0.015)	0.012 (0.009-0.018)	0.013 (0.010-0.018)	0.012 (0.009-0.016)
**PC 16:0_18:3**	0.002 (0.001-0.008)	0.002 (0.001-0.003)	0.002 (0.002-0.003)	0.002 (0.001-0.002)
**PC 16:0_18:3**	0.002 (0.001-0.003)	0.002 (0.001-0.004)	0.002 (0.001-0.003)	0.002 (0.001-0.004)
**PC 16:0_18:2**	0.041 (0.028-0.099)	0.038 (0.026-0.054)	0.039 (0.026-0.054)	0.038 (0.029-0.052)
**PC 16:0_18:1**	0.129 (0.089-0.198)	0.130 (0.106-0.174)	0.136 (0.110-0.174)	0.126 (0.106-0.158)
**PC 16:0_18:0**	0.010 (0.005-0.014)	0.010 (0.007-0.017)	0.010 (0.007-0.017)	0.010 (0.007-0.014)

Values are geometric mean (minimum – maximum). Molecular PC lipids are expressed as relative ratios of the total PC lipid signal.

PC: phosphatidylcholine, number of carbons:number of double bonds.
